# Confounding dynamic risk taking propensity with a momentum prognostic strategy: the case of the Columbia Card Task (CCT)

**DOI:** 10.3389/fpsyg.2015.01073

**Published:** 2015-08-07

**Authors:** Łukasz Markiewicz, Elżbieta Kubińska, Tadeusz Tyszka

**Affiliations:** ^1^Centre for Economic Psychology and Decision Sciences, Kozminski UniversityWarsaw, Poland; ^2^Cracow University of EconomicsCracow, Poland

**Keywords:** Columbia Card Task (CCT), dynamic risk taking, prognostic strategies, positive and negative recency, streaks perception

## Abstract

Figner et al. (2009) developed the Columbia Card Task (CCT) to measure risk-taking attitudes. This tool consists of two versions: in the COLD version the decision maker needs to state in advance how many cards (out of 32) they want to turn over (so called static risk taking), in the HOT version they have the possibility of turning over all 32 cards one-by-one until they decide to finish (dynamic risk taking). We argue that the HOT version confounds an individual’s willingness to accept risk with their beliefs in trend continuation vs. trend reversal in a prognostic task. In two experimental studies we show that people believing in trend continuation (momentum subjects) turn over more cards than those believing in trend reversal (contrarians) in the HOT version of the task. However, this is not the case in the COLD version. Thus, we provide evidence that, when considered as a dynamic risk propensity measure, the number of turned over cards in the HOT version of the CCT is a contaminated measure and reflects two phenomena: (1) risk preference and (2) the decision-maker’s belief in trend continuation. We speculate that other dynamic risk taking measures can also be biased by a momentum strategy.

## Introduction

Traditional static risk taking measures such as that of Holt and Laury (2002) have limited success in predicting individual differences in naturalistic risk-taking (Wärneryd, 1996; Kubińska and Markiewicz, 2012a,b) Therefore dynamic risk taking measures have been introduced (Wallsten et al., 2005). Dynamic risk taking occurs in situations where a decision maker faces recurring risky opportunities and where “outcome feedback at one point in time provides information that alters the subjective event probability prior to the next opportunity” (Wallsten et al., 2005, p. 863). Many authors argue that such measures are much better predictors than static measures of real-life risk taking behaviors such as drinking, smoking, or stealing (Lejuez et al., 2003, 2007). The HOT version of the Columbia Card Task (CCT; Figner et al., 2009) is one such dynamic risk taking measure (Dahne et al., 2013). The main aim of this study was to test whether the task confounds measurement of risk propensity with individual beliefs in trend continuation.

In each of 64 CCT^1^ rounds, participant (P) sees a deck of 32 loss and gain cards face down and characteristics of particular round: the number of loss cards (n) hidden among all remaining gain cards (32-n), the monetary amount associated with each loss card, and the amount associated with the gain cards. P’s task is to turn over cards to achieve a total gain as big as possible at the end of the last round. What P does not know is that only nine rounds of the game are generated by chance. The remaining 54 rounds are rigged to let the respondents turn over 32-n cards with no loss card appearing. Only these rigged feedback rounds are used as an indicator of risk preference (measured as the average number of turned over cards in all rigged 54 rounds).

Two versions of the CCT are available – HOT and COLD (for dynamic and static risk taking measurement respectively). Contrary to the HOT task that provides win/loss feedback after each card is turned over and feedback on number of points after each single round, the COLD task only provides points feedback when P completes the entire task, all 63 rounds. In each round of the HOT task, P points to a face-down card to turn it over and to see its face. If the card is a gain card (a smiling face), the gain is added to the total game balance, and then P points to the next card. The situation involves dynamic risk taking since each turned over gain card makes finding a loss card more probable by restricting the pool of gain cards among a fixed number of loss cards. P can turn over cards until they decide that the risk of turning over the next card is too high *or* until they encounter the loss cards. In the COLD task, however, P does not point to particular cards, but needs to decide in advance how many out of the 32 cards to turn over in the particular round that is described by the number of loss cards, loss amount, and gain amount (thus it relates to static risk taking) Furthermore, P knows that a draw will be made by the computer after they complete the entire task of 63 rounds.

As shown by Loewenstein et al. (2001) immediate feedback increases arousal on a task. By introducing immediate feedback to the CCT HOT version the authors made it feasible to track dynamic risk taking propensity, while the CCT COLD version with no feedback makes it feasible to track static propensity. Consequently Figner et al. (2009) demonstrated that the HOT condition is associated with higher electro-dermal activity than the COLD condition. This supports the authors’ suggestion that deliberative, cognitive processes prevail in the COLD condition, while affective processes prevail in the HOT one.

The authors of the CCT point out that far more cards are turned over in the HOT than in the COLD condition. Therefore the question arises as to what may encourage a decision maker to turn over more cards in the HOT task than the COLD task. We believe that the number of cards turned over in the HOT version (used as a measure of risk preference) is in fact a confounded measure of two phenomena: risk taking and the human propensity to follow trends. The CCT HOT task encourages a very specific propensity: the great majority of rigged rounds (54 out of 63) include positive feedback, therefore subject can if fact safely turn 32-n cards in each of the rigged rounds, getting positive feedback after turning each following cards (it is like throwing a long sequence of wins when throwing two sided coin with win and lose side). In several studies of animals and humans it has been shown that individuals adopt the so called win–stay/lose–shift (WSLS) strategy (Imhof et al., 2007; Wilke and Barrett, 2009; Blanchard et al., 2014). This tendency may account for the higher number of turned over cards in the HOT condition compared to the COLD condition. At the same time, it suggests possible contamination of the HOT task by two factors: risk propensity and following the WSLS.

Indeed, when individuals observe a series of events in the real word, they form strong expectations about the next event. Research on judging sequences of binary events [see the overview in Oskarsson et al. (2009)], both random and nonrandom, has shown that some individuals have expectations that a streak of events will continue (so called positive recency, momentum or “hot hand” beliefs), while others at the same time subjected to the same stimuli seem to believe in trend reversal (contrarian, negative recency propensity, and gambler’s fallacy). In these studies (e.g., Ayton and Fischer, 2004; Gronchi and Sloman, 2008; Tyszka et al., 2008; Kubińska and Markiewicz, 2012c; Kubińska et al., 2012), Ps observing a sequence of events are asked to make predictions about the next event. Thus, their strategy for forecasting uncertain events can be ascertained.

We suspected that rigged rounds feedback in the HOT condition would encourage momentum followers (positive recency) to collect more cards than contrarians (who would expect trend reversal and reveal a negative recency effect). Thus the CCT HOT condition is not a pure risk propensity measurement, but also measures an individual’s prognostic strategy. Thus, the higher number of turned over cards by momentum followers in the HOT condition (as compared to contrarians) does not reflect their higher risk preference but reflects task bias. In short, the CCT HOT version catches not only risk taking propensity but also the “hot hand” beliefs (Gilovich et al., 1985; Ayton and Fischer, 2004; Burns and Corpus, 2004) popular in sports, which refers to the conviction that a player has a higher chance of making a shot after two or three successful shots (resulting in “streaks”). In the same manner, by participating in many rigged feedback rounds, the positive feedback sensitive individual (momentum decision maker) can start to believe that they can turn over more cards because they have a lucky (hot) hand, and or are on a streak. Thus we hypothesized that, **H1: Momentum decision makers should turn over more cards in the CCT HOT condition than contrarians**. However, no such difference was expected in the COLD condition.

## Method – Study 1

Study 1 was conducted to verify the H1 hypothesis, which states that momentum decision makers would turn over more cards in the CCT HOT condition than contrarians.

### Participants and Procedure

Ps were students of Cracow University of Economics, *N* = 256 participants (mostly females: 86%, age *M* = 24.46 years, SD = 5.32). They gave their informed consent in accordance with the APA Ethical Principles of Psychologists and Code of Conduct. The study was approved by Kozminski University ethics committee and at the end of the study participants were fully debriefed. Ps performed the study individually, in front of a computer screen separated by cubicles from other PC stations, providing privacy from other participants. The duration of the whole procedure amounted to 42 min on average.

### Measures

The experiment was run using Inquisit by Millisecond Software. The prognostic strategy task was programed by the first author, while the CCT script was downloaded from the Inquisit Task Library (http://www.millisecond.com/download/library/).

#### Prognostic Strategy

**FIGURE 1:**
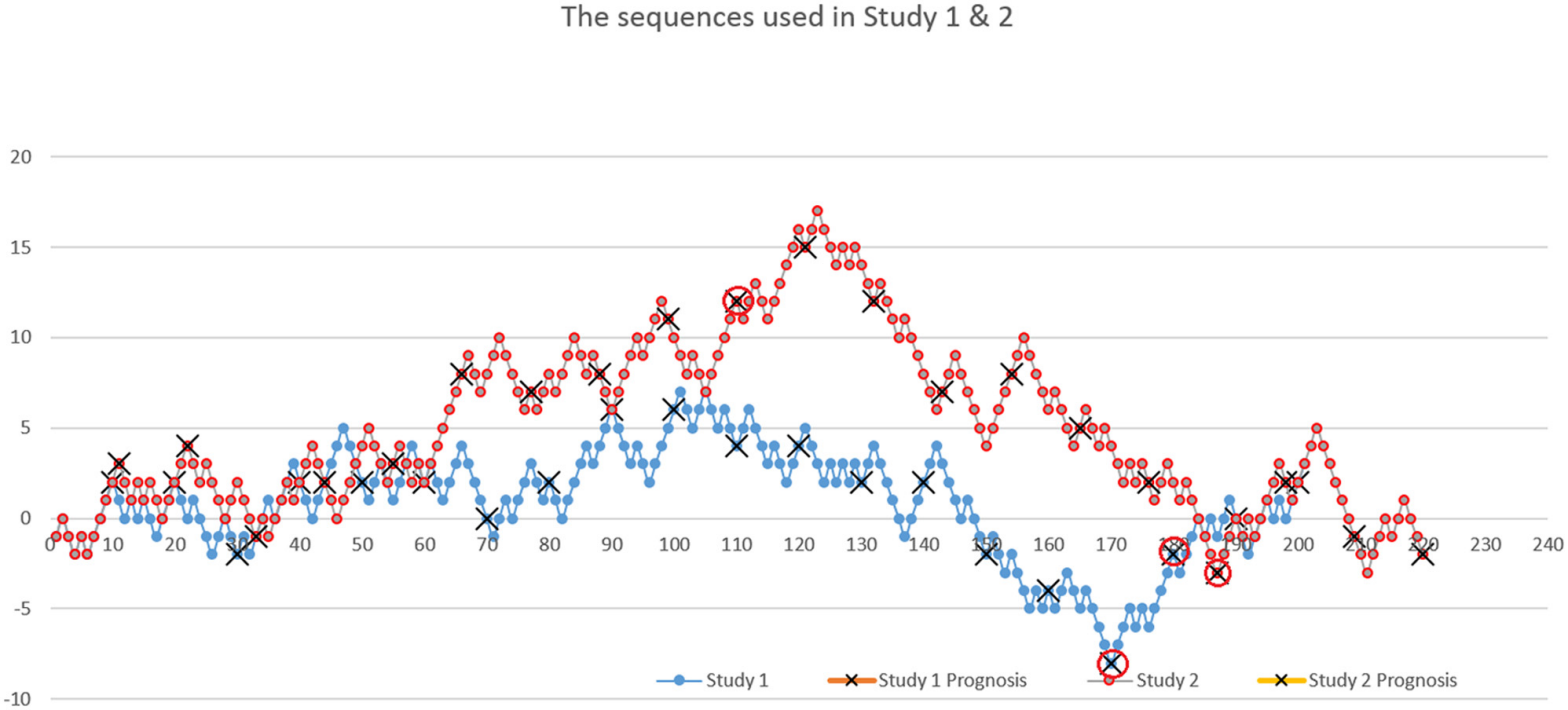
**The sequence presented to participants.** Tails are coded as -1 and heads as +1 (Studies 1 and 2). Participants saw separate events one after another, and not the whole history as presented in the chart. Red circles highlight the critical decisions for the longest sequences determining prognostic strategy.

Prognostic strategy use was measured by a procedure used by Tyszka et al. (2008), often referred to as a recency test. Ps were asked to make predictions based on observation of a randomly generated sequence of binomial events (a two-point distribution with equally probable values). The task instructions informed Ps that a sequence of fair coin tosses (with equal probabilities of head and tails) was going to be presented on the screen. The participants were asked to observe 20 series of 10 coin tosses (200 tosses in total). At every 10th event, Ps had to make a prediction about the next event. Participants’ aims were to make as many correct predictions as possible. The sequence of binomial events for Study 1 with circled predicted events is presented in **Figure 1**.

Ps were classified *post hoc* into two groups: momentum (M) and contrarian (C) decision makers, based on two crucial rounds ending with the longest streak lasting for three events – round 17 ending with the streak of three heads and round 18 ending with the streak of three tails (circled points in **Figure 1**). Respondents anticipating trend continuation in both aforementioned rounds were classified as momentum followers (*n* = 48), and those predicting trend reversal in both rounds as contrarians (*n* = 110), leaving *n* = 98 respondents unclassified^2^. All other rounds with no streaks or streaks of two events were discarded in the analysis as not being perceived as streaks by respondents (Carlson and Shu, 2007) in line with previous studies (Tyszka et al., 2008). The *post hoc* control analysis revealed that the M and C groups were balanced in terms of gender (*p* > 0.05) however, contrarian group members were slightly older (*M* = 24.34 years) than momentum members (*M* = 23.10), *t*(149.762) = 2.146, *p* < 0.05.

#### The Columbia Card Task

After completing the recency test, Ps were randomly assigned to one of the two conditions, taking the CCT either in the COLD (*n* = 127, 84% females) or HOT (*n* = 129, 87% females) version. The control analysis revealed that participants were balanced in terms of age and gender (*p* > 0.05). On ending, participants gave socio-demographic information and were debriefed.

### Results – Study 1

**FIGURE 2:**
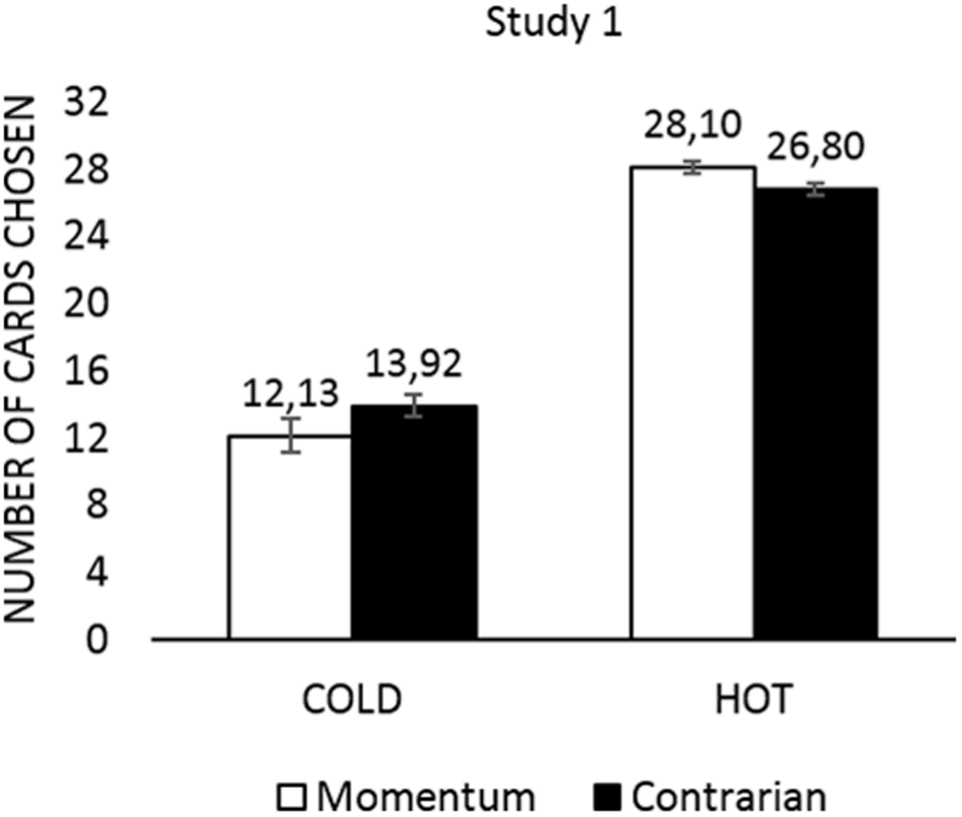
**Study 1: the number of turned over cards in the HOT and COLD conditions separately for momentum and contrarian participants**.

On average, respondents taking part in the HOT task^3^ disclosed more cards (*M* = 26.95; SD = 3.17) than those taking part in the COLD task (*M* = 13.24; SD = 4.59), as revealed by *t*-test, *t*(223.669) = 27.743, *p* = 0.001; *d* = 3.48. To test the hypothesis (**H1**) that prognostic strategy, as measured externally for the CCT, influences the number of turned over cards in the HOT condition, a two-way independent factorial ANOVA was conducted with the results presented in **Figure 2**. We demonstrated a significant main effect of CCT task on the number of turned over cards, *F*(1,154) = 506.71, *p* = 0.001, η^2^ = 0.767. There was also a significant interaction effect of CCT task and prognostic strategy on the number of turned over cards, *F*(1,154) = 5.18, *p* = 0.024; η^2^ = 0.033. Prognostic strategy influenced the number of turned over cards differently in the COLD and HOT tasks. The observed interaction contributes to better understanding of the CCT HOT task, since such interactions are nowadays considered to provide major contributions to judgment and decision making studies (Appelt et al., 2011).

As expected, in the HOT condition momentum followers turned over more cards than contrarians, *t*(80) = 2.034, *p* = 0.045, Cohen’s *d* = 0.494, while the number of turned over cards for contrarians and momentum followers did not differ significantly for the COLD condition, *t*(74) = 1.430, *p* = 0.157, *d* = 0.353. Thus, momentum followers turned over more cards than contrarians in the HOT condition but not in the COLD condition. However, the effect size in the HOT task was only moderate.

## Study 2

Study 2 was conducted to test the robustness of the Study 1 results and to verify the strength of the Study 1 effect. Here, we included another sequence in the recency task to ensure results were not dependent upon the specific sequence in Study 1. Also, we aimed to recruit a more diversified sample in terms of gender (Study 1 involved a predominantly female sample). Since the Study 2 research hypothesis related only to the HOT CCT condition, the COLD task was not utilized here.

### Participants and Procedure

Sixty-five students of Kozminski University, mostly females (60%), age *M* = 22.53 years (SD = 5.23), took part in the research after giving their informed consent in accordance with the APA Ethical Principles of Psychologists and Code of Conduct. The study was approved by Kozminski University ethics committee and at the end the study participants were fully debriefed. The participants performed the study individually, in front of a computer separated by cubicles from other PC stations, providing privacy from other participants.

### Measures

The Study 1 procedure (with the whole group performing CCT HOT only) was repeated, however, we used a new randomly generated sequence of binary events to ensure that results were not dependent on the specific sequence used in Study 1. The new sequence is graphically presented in **Figure 1**. Ps made their prognosis at every 10th event, and were classified into momentum and contrarian groups based on their decisions in the longest rounds (11th – ending with the streak five heads, and 19th – ending with the streak five tails). In this way we obtained a group of *n* = 9 contrarians (67% females) and *n* = 18 momentum followers (72% females), leaving *n* = 38 unclassified (53% females). The *post hoc* control analysis revealed that the M and C groups were balanced in terms of gender and age (*p* > 0.05). At the end of the procedure participants completed socio-demographic questionnaires.

### Results – Study 2

**FIGURE 3:**
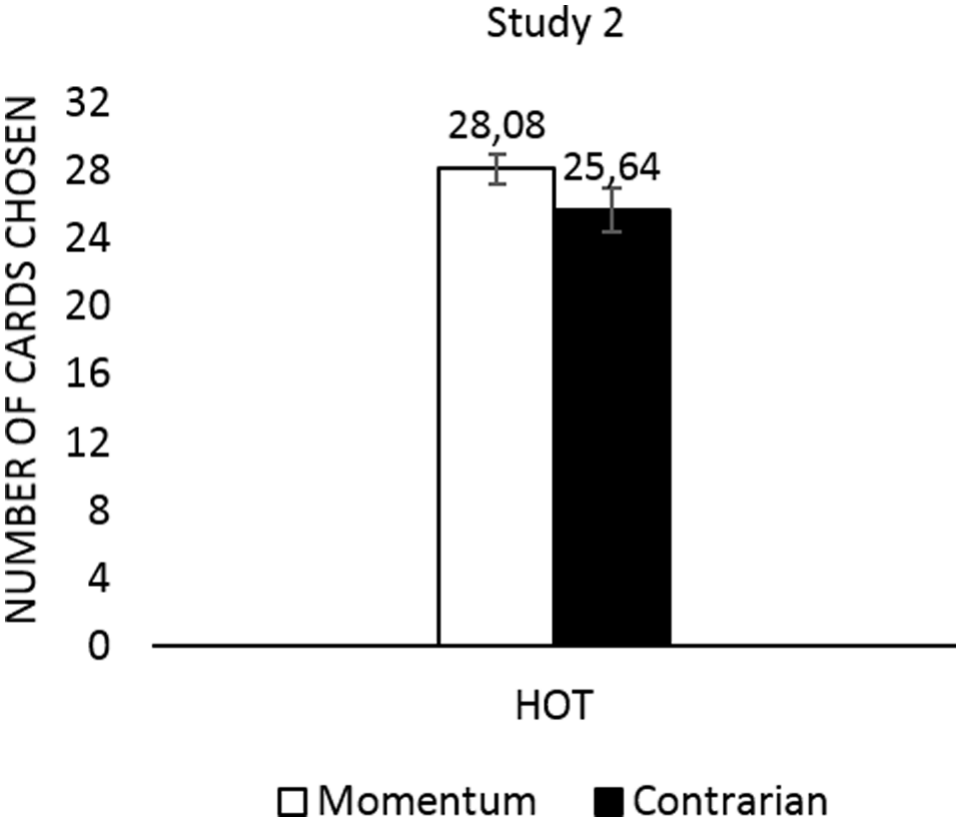
**Study 2: the number of turned over cards in the HOT CCT separately for momentum and contrarian participants**.

In Study 2 we retested hypothesis **H1**, assuming that prognostic strategy, as measured externally for the CCT, would influence the number of turned over cards in the HOT condition. We assumed that in the HOT condition the momentum followers would turn over more cards than the contrarians. To validate the results of Study 1 *t*-test was conducted which showed that momentum followers turned over more cards than contrarians [*t*(25) = 2.270, *p* = 0.032; *d* = 0.927]. The result (**Figure 3**), with large effect size this time, therefore provides additional support for the Study 1 results and adds significantly to research using other recency task. The difference between Studies 1 and 2 was the different event sequence used in the recency test (see **Figure 1**), and therefore the results show that verification of H1 does not depend on the sequence used to divide people into contrarian and momentum groups.

## General Discussion

### Testing of the Hypothesis

Studies 1 and 2 both supported the hypothesis that in the CCT HOT condition momentum decision makers would turn over more cards than contrarians. No such difference was observed for the COLD condition. Thus, it can be concluded that the HOT version of the CCT confounds risk attitudes and prognostic strategy use. We believe that momentum followers, who believe in the continuation of streaks of positive events, simply tend to use the WSLS strategy more frequently than participants who do not believe in the continuation of streaks. Also, the HOT task guarantees participants long streaks of positive events (gain cards). Thus, those believing in streak continuation (momentum followers) turn over more cards when a streak appears than those believing in streak reversion (contrarians) who stop the game.

### Theoretical Implications

One can speculate that a belief in the continuation of a streak of events can also influence responses to other dynamic risk taking tools, such as the Balloon Analogue Risk Task (Lejuez et al., 2002; Dahne et al., 2013; Lauriola et al., 2014), the Devil’s Task (aka Slovic’s Risk Task –(Slovic, 1966) and others (Gardner and Steinberg, 2005; Fernandez-Duque and Wifall, 2007; Pleskac, 2008). It is a general characteristic of dynamic risk taking tasks that they allow repeated choices with gradually rising small probabilities of failure. Thus, participants experience a sequence of events, usually a long sequence of positives, before experiencing a failure (if this is experienced at all). As suggested by Figner et al. (2009), such a dynamic is an important characteristic of real-world risk-taking (e.g., driving a number of extra miles without refueling when a fuel light flashes). The question therefore arises as to whether only certain dynamic risk taking measures (e.g., the CCT HOT task) confound risk with type of prognostic strategy use, or alternatively whether the whole concept of dynamic risk taking (Weber and Johnson, 2008) confounds these two phenomena. The further studies should investigate other dynamic risk taking tools to test whether they assign higher risk taking scores to momentum followers than to contrarians.

### An Alternative Explanation

The results demonstrated that in the HOT condition momentum followers turned over more cards than contrarians. We have explained this by referring to the momentum characteristics of the CCT HOT task. An alternative explanation might be that momentum followers are willing to accept higher risks than contrarians. However, if anything, contrarians should reveal higher risk taking propensities than momentum followers (Kubińska and Markiewicz, 2012c; Marmurek et al., 2013). In a sequence of binomial events appearing with the same probability, momentum followers treating the last event as a reference point could display a status quo effect (opting for trend continuation), while contrarians might either adopt a different reference point, or, as less risk averse individuals, not opt for the status quo and take an action which does not follow the trend. Previous research has demonstrated that contrarian investors create more risky portfolios in terms of SD and variance (Kubińska and Markiewicz, 2012c), thus it remains possible that M-C differences in risk taking propensity exist only in the cold domain, dominated by calculated risk taking. However, our study hasn’t detected any significant M-C difference in cold conditions, although the directional change favors the hypothesis of higher risk taking propensity among contrarians. Further research should investigate if, and when, contrarians take more risk than momentum followers.

### Limitations

There are some possible limitations of our research. The momentum and contrarian groups contained roughly half of the subjects each. Therefore the claim that the CCT HOT task confounds risk propensity with prognostic strategy use only applies to the part of any statistical population that could be assigned to M or C groups: the confounding nature of the HOT task is relevant only to the part of a population that could be assigned to such groups and is not relevant to the part of any population that is not sensitive to trends. The fact that only half of the sample could be classified as M or C may result from either such a structure existing in the present population or from shortcomings of currently used methods for M and C classification. The currently used method of measuring prognostic strategy use (Tyszka et al., 2008) requires respondents to make many choices. However, only two choices related to the longest sequences are used to assign participants to momentum or contrarian groups. This makes the recency test a noisy tool, potentially misclassifying subjects (someone not tracking the sequence can be wrongly misclassified as M or C with *p* = 0.5). Nevertheless, even using a noisy classification tool our set of experiments supported our hypothesis. This makes us believe that a better classification tool would provide even clearer evidence in favor of the hypothesis tested. Further studies should also address the limitations of the current method of investigating prognostic strategies. We believe that further research could use the Hierarchical Bayes method (Scheibehenne and Studer, 2014) to classify decision makers based on a whole set of decisions. In the “drift model” introduced by Scheibehenne and Studer (2014), the probability of predicting the same outcome for the next event to be observed is related to the length of the last run. If there is a positive relationship between the probability of the trend continuation and the run length, then those respondents are more likely to predict the continuation of a streak and they are classified as momentum maintainers. A negative relationship between the probability of the trend continuation and the run length indicates that probability of predicting the same outcome decreases with the run length and those respondents are classified as contrarians. In the Hierarchical Bayesian approach, the prognostic strategies classification is made based on the sensitivity of the respondents to the whole trend, rather than being based on only a few arbitrary moments.

## Author Contributions

All authors contributed equally to the manuscript.

## Conflict of Interest Statement

The authors declare that the research was conducted in the absence of any commercial or financial relationships that could be construed as a potential conflict of interest.

## Footnotes

1 Listed in the Decision Making Individual Differences Inventory (DMIDI) database under the link: http://www.sjdm.org/dmidi/Columbia_Card_Task.html (official webpage: http://www.columbiacardtask.org).

2 Leaving respondents unclassified is intended and planned. The previous studies (Odean, 1999) reveal that there is a group amounting to 50% of samples which takes streaks into consideration while making prognoses. It seems that the other half are insensitive to streaks (Scheibehenne and Studer, 2014).

3 The number of turned over cards in our HOT condition participants is higher compared to the original Figner et al. (2009) study. However, the number is similar to that for the group of respondents aged 17–19 in the same study. We believe that gender, educational profile and cross cultural differences play a role here.
